# Phage adsorption and lytic propagation in *Lactobacillus plantarum*: Could host cell starvation affect them?

**DOI:** 10.1186/s12866-015-0607-1

**Published:** 2015-12-02

**Authors:** Mariángeles Briggiler Marcó, Jorge Reinheimer, Andrea Quiberoni

**Affiliations:** Instituto de Lactología Industrial, Facultad de Ingeniería Química, Universidad Nacional del Litoral, Santiago del Estero 2829, 3000 Santa Fe, Argentina

**Keywords:** Lactic acid bacteria, Cell starvation, Bacteriophage, Phage propagation

## Abstract

**Background:**

Bacteriophages constitute a great threat to the activity of lactic acid bacteria used in industrial processes. Several factors can influence the infection cycle of bacteriophages. That is the case of the physiological state of host cells, which could produce inhibition or delay of the phage infection process. In the present work, the influence of *Lactobacillus plantarum* host cell starvation on phage B1 adsorption and propagation was investigated.

**Result:**

First, cell growth kinetics of *L. plantarum* ATCC 8014 were determined in MRS, limiting carbon (S-N), limiting nitrogen (S-C) and limiting carbon/nitrogen (S) broth. *L. plantarum* ATCC 8014 strain showed reduced growth rate under starvation conditions in comparison to the one obtained in MRS broth. Adsorption efficiencies of > 99 % were observed on the starved *L. plantarum* ATCC 8014 cells. Finally, the influence of cell starvation conditions in phage propagation was investigated through one-step growth curves. In this regard, production of phage progeny was studied when phage infection began before or after cell starvation. When bacterial cells were starved after phage infection, phage B1 was able to propagate in *L. plantarum* ATCC 8014 strain in a medium devoid of carbon source (S-N) but not when nitrogen (S-C broth) or nitrogen/carbon (S broth) sources were removed. However, addition of nitrogen and carbon/nitrogen compounds to starved infected cells caused the restoration of phage production. When bacterial cells were starved before phage infection, phage B1 propagated in either nitrogen or nitrogen/carbon starved cells only when the favorable conditions of culture (MRS) were used as a propagation medium. Regarding carbon starved cells, phage propagation in either MRS or S-N broth was evidenced.

**Conclusions:**

These results demonstrated that phage B1 could propagate in host cells even in unfavorable culture conditions, becoming a hazardous source of phages that could disseminate to industrial environments.

## Background

As acknowledged, lactic acid bacteria (LAB) are widely used in the dairy industry, being responsible for carrying out the fermentation process in the production of lactic acid from lactose. In addition, LAB contributes to food preservation as well as the development of flavor and texture of the final products [1].

However, the activity of LAB can be threatened by bacteriophages. Phage infections constitute a serious problem in the fermentative dairy industry since they can generate a delay or a complete inhibition of milk acidification leading to a partial or total loss of the product. Therefore, phage attacks exert a great impact on technological and economic aspects in dairies [2]. Consequently, several strategies have been designed in order to control the episodes linked to phage attacks. Direct vat inoculation of starter cultures, culture rotation programs, use of starter cultures with increased phage resistance, adequate factory design, and optimized sanitation are some control strategies against phage dissemination in industrial environments [3]. Nevertheless, and taking into account that none of the mentioned schemes are completely effective, a combination of them are usually applied in industrial environments to improve the control of phage infection.

As it is known, phage propagation depends on the physiology of the host cells [4–6]. Thus, alterations in physiological state of bacteria could produce changes in the host cell’s susceptibility to phage infections and/or the productivity of the phage infection [7]. However, some authors reported the production of phage progeny even when the growth conditions of the bacterial strain were unfavorable [8–10]. In this regard, it should be noted that the culture growth conditions in natural environments are quite different from those found in the laboratory, in which bacteria have all factors essential for a favorable growth [10]. By way of example, the ability of a *Streptococcus thermophilus* phage to penetrate into a biofilm and initiate the infection cycle was evidenced [11]. Therefore, bacterial cells that remain on equipment surface and pipes after manufacture and cleaning processes could help phage propagation and the subsequent dispersion of phages to industrial environments. As a result, analysis of the ability of phages to propagate in bacterial cells which are not in their optimum growth conditions are necessary. Much research related to inhibition of phage development when cell starvation conditions are induced was carried out mainly on *Escherichia coli* [12, 13]. Regarding LAB, the available information is only focused on the influence of bacterial metabolism (studied using starved cells) on some stages of the infective cycle (phage adsorption/DNA injection) in *Lactobacillus casei* [14, 15].

In the present study, the influence of cell starvation conditions on phage propagation was studied. To carry out the assays, a virulent phage ATCC 8014-B1 (herein referred to as B1) was chosen due to its simple handling in the laboratory. Furthermore phage B1 was previously studied in depth [16–18], including its complete molecular characterization [19]. Its host strain *Lactobacillus plantarum* ATCC 8014 also evidence probiotic potentiality [20].

The aim of the present work is to investigate the ability of *L. plantarum* phage B1 to propagate in bacterial cells under starvation conditions.

## Results and discussion

### Bacterial growth kinetics

**Fig. 1:**
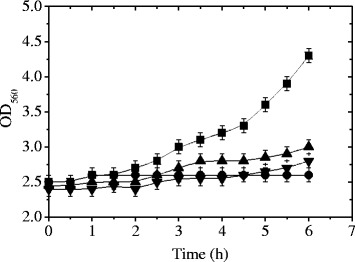
Growth kinetics of *L. plantarum* ATCC 8014 under starvation conditions. The experiments were carried out at 37 °C in MRS (■, control), S (●), S-N (▲) or S-C (▼) broth. The values are the mean of two determinations

When *L. plantarum* ATCC 8014 was subjected to starvation conditions (in S, S-C or S-N broth), reduced growth rates were evidenced in comparison to those reached in MRS (Fig. 1). Bacterial cells were able to grow until a OD_560_ value of 4.3+/−0.4 in MRS after 6 h at 37 °C whereas OD_560_ values of 3.0+/−0.2 (in S-N broth) and 2.8+/−0.3 (in S-C broth) were observed. *L. plantarum* ATCC 8014 strain was not able to grow in S broth since OD_560_ values around 2.6+/−0.3 were observed even after 6 h of incubation (Fig. 1). A decreased μ_max_ was observed for bacterial strain growing in S-N, S-C and S broth (0.033+/−0.008, 0.026+/−0.007, 0.033+/−0.006 Δln OD/h, respectively) in comparison to that obtained in MRS broth (0.090+/−0.011 Δln OD/h). No significant differences (*p* < 0.01) were observed for bacterial strain under starvation conditions (S-N, S-C and S broth).

### Adsorption test

As it is well known, the first step in phage infection is the adsorption on the host cell surface. This stage depends on the presence of specific attachment sites (phage receptors) on the cell wall surface [17]. In the present work, more than 99 % of phage particles were adsorbed on *L. plantarum* ATCC 8014 strain after 30 min in all the media studied (MRS, S, S-C, S-N broth). Similar results were observed when cells were previously starved during 17 h and subsequently assayed for phage adsorption. According to these results, the first stage of phage infection occurs even if host cells are previously subjected to starvation conditions. A similar behavior was observed in *E. coli* since phage MS2 adsorbed efficiently on starved cells [12]. In concordance with these results, several authors verified that adsorption is an energy-independent process. In a previous work, phage B1 was demonstrated to be able to adsorb on viable but non-proliferating cells (cells treated with antibiotics to inhibit the protein synthesis) [17]. On the other hand, phage PL-1 (*L. casei*) was able to adsorb on cells killed by being kept or exposed to UV light [21]. Regarding to Gram-negative bacteria, energy requirement for phage adsorption process were dissimilar. Adsorption was independent on energy for phages T4 and fd (*E. coli*) [22], [23]. On the contrary, Daugelavicius et al. [24] reported an energy-dependent process in *Salmonella typhimurium* (phage PRD1).

Although an energy-independent process would be expected for phage binding, the subsequent steps in the life of a phage need energy to be completed. In this regard, the energy requirement to carry out some stages of the lytic cycle (DNA injection) was previously studied in lactic acid bacteria. Specifically, the presence of intracellular high-energy compounds was demonstrated to be indispensable for penetration of phage PL-1 genome into the bacterial host cell (*L. casei*) [14, 15, 21, 25]. In addition, phage PL-1 adsorbed on starved cells but the DNA injection into the host cell was inhibited due to the absence of an active cell metabolism (decrease of intracellular ATP content, inhibition of protein synthesis). Then, phage PL-1 was able to complete the injection of their genome when the ATP content was restored to the original state [15].

### Influence of cell starvation on one-step growth curve

#### Post-infection starvation

**Fig. 2:**
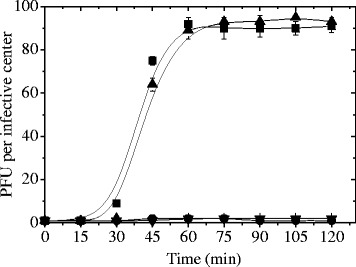
Influence of bacterial cell starvation on one-step growth curve. *L. plantarum* ATCC 8014 cells grown in MRS, infected with phage B1 and then resuspended in MRS (■), S (●), S-N (▲) or S-C (▼) broth for one-step growth curve. The values are the mean of two determinations

The ability of phage B1 to propagate in *L. plantarum* ATCC 8014 strain was studied in media devoid of carbon (S-N), nitrogen (S-C) or carbon/nitrogen (S) sources. In this sense, deprivation of carbon source did not inhibit lytic development of the phage B1 which was able to propagate in bacterial cells resuspended in S-N broth (Fig. 2). As for this starvation condition, burst size value (90 PFU per infective center) and latent period (30 min) were similar to those observed in MRS (Fig. 2). In contrast, formation of phage progeny was inhibited in infected cultures devoid of the nitrogen (S-C broth) and carbon/nitrogen (S broth) sources (Fig. 2). Although yeast extract, meat extract and peptone supply carbohydrate compounds, its concentration would be depreciable in comparison to that provided by glucose (final concentration of 2 % w/v in MRS broth). In consequence, the inhibition of phage production observed in S-C medium would be mainly due to the nitrogen compounds deprivation. To our knowledge, this would be the first study related to the influence of cell starvation on phage propagation in lactic acid bacteria. However, this thematic was studied in *E. coli* phages. In this sense and in concordance with our results, it was demonstrated *E. coli* phages were not able to propagate in starved cells in a mineral salt medium [13]. In particular, some authors reported phage T4 is able to adapt its multiplication cycle according to the host cell physiological state. Thus, the lytic cycle might be prolonged (rate of phage release and burst size decreased, eclipse and latent periods increased) when the growth rate of host strain decreased [5, 10]. In a previous work, Golec et al. [8] evidenced the presence of two T4 phage particle subpopulations under starvation conditions: those that can infect starved cells and those that are not able to do. These subpopulations have differences in their ability to adsorb on bacterial cells. So, phage T4 would prevent it adsorption on host cells by retracting its tail fibers and it would be a reaction against unfavorable conditions of cell growth. In a subsequent work, Golec et al. [9] reported the involvement of phage-encoded proteins (RI and RIII) in regulation mechanisms playing when phage infection happen on under slowly growing bacterial cells.

On the other hand, some authors reported the generation of a specific metabolic response (stringent response) under starvation conditions which is characterized by production of high level of a nucleotide (alarmone) called ppGpp [8, 26]. Increased levels of ppGpp alarmone would generate a decreased rate of protein synthesis, thus limiting the DNA replication as well as transcription of many genes due mainly to interactions with RNA polymerase [26, 27]. Nowicki et al. [26] linked the stringent response to phage progeny production in amino acid-starved cells of *E. coli*. Under this condition, ppGpp would limit both the lytic development and phage DNA replication. In a similar way, and regarding to lactic acid bacteria, a stringent response mediated by ppGpp generated under stress conditions was reported for *Lactococcus lactis* and *Lactobacillus* but it was not linked to phage propagation [27, 28].

On the other hand, Wegrzyn et al. [29] suggest that elevated ppGpp levels could have influence on “lysis versus lysogenization” decision in *E. coli*. Besides, the connection between lysogenization and the cAMP (nucleotide), which is related to the intracellular energy contents, was reported [30]. In this sense, cAMP determines the stability of CII protein, which represses the transcription of lytic promoters in benefit of lysogenic cycle. Under starvation conditions, a high concentration of cAMP produces the CII protein stabilization. On the contrary, when bacterial cells have high energy concentration, its intracellular cAMP content is low and in consequence the levels of lysogenization are reduced due to the proteolysis of CII protein [30].

**Fig. 3:**
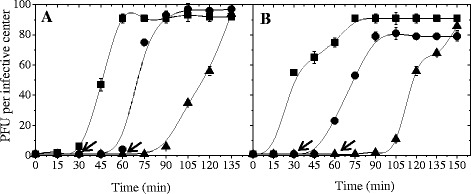
Influence of bacterial cell starvation (post-infection) on one-step growth curve. *L. plantarum* ATCC 8014 cells grown in MRS, infected with phage B1 and then resuspended in S (**a**) or S-C (**b**) broth for one-step growth curves. At 30 (●) or 60 (▲) min, the limiting compounds were added. Control one-step curves in MRS (■). Arrows indicate time at which the appropriate compounds were restored. The values are the mean of two determinations

**Fig. 4:**
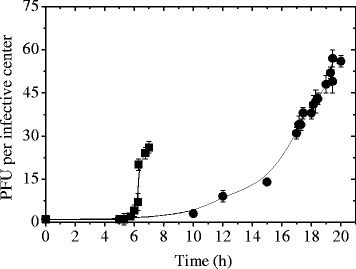
Influence of bacterial cell starvation (post-infection) on one-step growth curve. *L. plantarum* ATCC 8014 cells grown in MRS, infected with phage B1 and then resuspended in S broth for one-step growth curves. At 5 (■) and 17 h (●), the limiting compounds were added. Arrows indicate time at which the appropriate compounds were restored. The values are the mean of two determinations

On the other hand, the effect of the restoration of the optimum culture conditions (MRS) on phage propagation was studied. Thus, the infected cultures initially devoid of nitrogen or glucose/nitrogen compounds were supplemented with the limiting substance/s at 30 min and 60 min of infection started (Fig. 3). In this regard, phage progeny production began 30 min after the appropriate compound was added, reaching similar levels to those obtained in MRS (Fig. 3). In the same way, bacteriophage replication was studied working with infected cultures starved for 5 and 17 h in S (devoid of carbon and nitrogen sources) broth (Fig. 4). In both cases, phage B1 was able to propagate when the appropriate substances (carbon and nitrogen) were restored. In particular, a few phage particles were released in S broth, during the starvation period of 17 h. However, phage propagation rate was higher when the limiting compounds were restored (Fig. 4). A hypothesis to explain this behavior could be related to the decrease of both OD_560_ values (from 1.488 to 1.358) and cell counts (from 3.0 × 10^8^ to 5.0 × 10^7^ CFU/ml) observed during the starvation period (17 h). In this sense, and due to the extreme starvation conditions to which cells are subjected for a long time, a partial autolysis of bacterial cells could occur. In consequence, phage particles formed using the remaining cell energy, could be released.

Similar results to those obtained in this work were evidenced in the case of *E. coli* phages. Thus, phage progeny production was restored by reintroduction of glucose to infected cultures of starved bacteria, irrespective of the length of the starvation period (up to 40 h of starvation) [13].

#### Pre-infection starvation

**Fig. 5:**
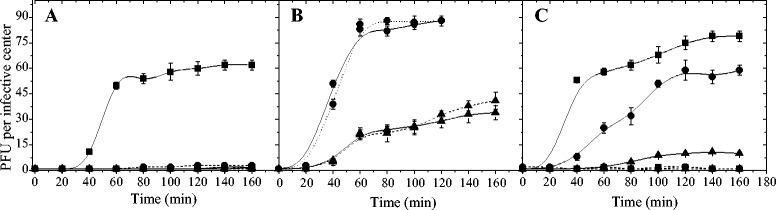
Influence of bacterial cell starvation (pre-infection) on one-step growth curve. *L. plantarum* ATCC 8014 cells grown in limiting nitrogen/glucose (**a**), limiting glucose (**b**), or limiting nitrogen (**c**) medium infected after 1 (■), 5 (●) and 17 h (▲) of starvation. One-step growth curve of phage B1 on starved cells, in MRS (—) or in the corresponding medium (------). The values are the mean of two determinations

The effect of the cell starvation conditions applied before phage infection was studied in S, S-N and S-C broth and for three starvation periods (1, 5 and 17 h) (Fig. 5). Phage B1 was able to propagate in cells previously starved in a medium devoid of carbon and nitrogen compounds (starvation period of 1 h) when the favorable culture conditions (MRS) were restored. On the contrary, phage progeny production was not evidenced in S broth. However, when starvation period was of 5 h phage development was completely inhibited either in MRS or S broth (Fig. 5, a). On the other hand, phage B1 completed it lytic cycle on bacterial cells that had been previously starved for carbon source (during 5 and 17 h). Development of phage B1 was similar in MRS and S-N broth for either starvation period (5 or 17 h of starvation). However, phage B1 evidenced a lower propagation rate on starved cells for 17 h. In this sense, burst size values were 90 and 40 PFU per infective center on starved cells for 5 and 17 h, respectively (Fig. 5, b). When cell cultures were starved in a medium devoid of nitrogen source (S-C broth), phage B1 propagation was achieved on starved cells for all the starvation periods assayed (1, 5 and 17 h) when the limiting substance (nitrogen) was restored. On the contrary, no phage replication was observed in S-C broth. In MRS, the phage propagation rate decreased as the starvation period was increased. So, the burst size values were 80 (1 h starvation), 60 (5 h starvation) and 10 (17 h starvation) PFU per infective center (Fig. 5, c). Props-Ricciuti [12] studied the phage propagation in starved cells infected with phage MS2 (*E.coli*). Under starvation conditions the production of phage progeny was observed though the phage particles were not released possibly due to failure in cell division. However, phage propagation and the subsequent cell lysis were observed when the favorable culture conditions were restored. On the contrary to what was observed for phage B1, MS2 phage release from nitrogen starved cells was not evidenced when the limiting substance was returned to the culture [12].

A dissimilar behavior related to phage B1 propagation was observed when the cell cultures were infected before or after the starvation period (in S broth during 5 and 17 h). When phage infection began before cell starvation, phage B1 completed the lytic cycle on *L. plantarum* ATCC 8014 strain in MRS. However, phage propagation was completely inhibited when bacterial cells were starved before phage infection. A similar behavior was observed in the case of *E. coli* phages [13]. The mechanism of impairment of phages to propagate on starved cells could be an adaptation strategy facing unfavorable environment conditions which could negatively affect its multiplication cycle [8]. Hence, through of these strategies, phages could remain in the environments while conditions for its propagation are not adequate and just propagate when those are favorable [10].

## Conclusions

Taking into account all the results obtained, phage B1 could propagate on bacterial cells even when the culture conditions are not favorable. In this sense, some interesting observations have arisen during regular monitoring of phages in industrial environments in our laboratory. That is to say that although bacteriophages were rarely found during the manufacture process or in the final products, their presence and persistence was evidenced in liquid effluents. Apart from lysogeny, that should never be ignored, phages could arise from a continuous propagation on residual bacterial cells coming from manufacture processes and then remaining in pipes or equipment containing the effluents, even when those cells are not in their optimal metabolic state. In consequence, this could constitute a potential and hazardous source of phages that could disseminate to the industrial environments.

## Methods

### Bacterial strains, phages and culture conditions

*L. plantarum* collection phage B1 [GenBank:JX486087] [19] and its sensitive strain *L. plantarum* ATCC 8014 were studied in this work. The host strain was maintained as frozen stock at −80 °C in MRS (Man Rogosa Sharpe) broth (Biokar, Beauvais, France) in the presence of 15 % glycerol, routinely reactivated overnight at 37 °C in MRS broth, and counted on MRS agar (48 h at 37 °C). MRS broth and MRS agar supplemented with 10 mmol l^−1^ CaCl_2_ (MRS-Ca) were used to propagate and count the phage B1. Phage stocks were prepared as described by Neviani et al. [31] and stored at 4 °C (MRS) and −80 °C (MRS broth added of 15 % v/v glycerol). Phage enumerations (PFU) were determined by the double-layer plaque titration method [32].

To carry out the experiments, MRS broth was prepared in the laboratory according to the following composition (in w/v): carbon source (glucose, 2 %); nitrogen source (peptone, 1 %; meat extract, 0.8 %; yeast extract, 0.4 %); polysorbate 80, 0.1 %; dipotassium hydrogen phosphate, 0.2 %; sodium acetate, 0.5 %; ammonium citrate, 0.2 %; magnesium sulfate, 0.02 %; manganese sulfate, 0.005 %. To reach starvation conditions, carbon compounds (S-N broth), nitrogen compounds (S-C broth) or carbon/nitrogen compounds (S broth) were removed. Note that yeast extract, meat extract and peptone are complex sources of nitrogen, amino acids, carbohydrates and vitamins. Although they provide some carbohydrates, this content is insignificant in comparison to that provided by glucose (final concentration of 2 % in MRS broth). In consequence, when yeast extract, meat extract and peptone are removed during one-step growth curve assays, the behavior observed would be mainly due to the nitrogen compounds deprivation.

### Preparation of cell cultures

For the methods described below (bacterial growth kinetics, adsorption tests and one-step growth curves), the host strain *L. plantarum* ATCC 8014 was grown in MRS (prepared in the laboratory) until exponential growth (OD_560nm_ = 0.5). Cells were then harvested and suspended in MRS, S-C, S-N or S broth, according to each experiment.

### Bacterial growth kinetics

To test the effect of starvation on the bacterial growth kinetics, cell cultures subjected to the treatment described previously (Preparation of cell cultures), resuspended in each medium (MRS, S-C, S-N or S broth) were incubated at 37 °C for 6 h. The optical density of the cell cultures was measured at 560 nm (OD_560_) and plotted against time. The maximum specific speed (μ_max_) was calculated for each bacterial growth curve as follows: μ_max_ = ln OD_f_ - ln OD_0_/Ɵ_f_ - Ɵ_0_, where OD_f_ is the final optical density; OD_0_ is the initial optical density; Ɵ_f_ is the final time; Ɵ_0_ is the initial time [33]. Aliquots of each medium in the absence of bacterial cells were assayed as a control. The R software (R Development Core Team, 2010, Boston, MA) was used for the statistical analysis. One-way ANOVA was utilized to analyze data using a general linear model procedure with Tukey pairwise comparison at 99 % confidence level.

### Adsorption test

To test the influence of a medium devoid of nutrients (carbon, nitrogen or carbon/nitrogen) on phage adsorption, cell cultures with the same treatment described previously (Preparation of cell cultures), were resuspended in MRS, S-C, S-N or S broth (1/2 of initial volume). Phages were added (multiplicity of infection, m.o.i = 1) and the mixtures incubated at 37 °C for adsorption during 30 min. Tubes were then removed and centrifuged (10,000 × g for 5 min) to sediment the phage-adsorbed bacteria. The supernatants were assayed for unadsorbed phages (double-layer plate titration), and the counts were compared with the titre of a control without cells. The results were expressed as percentages of the initial phage counts [17].

On the other hand, to test the influence of extreme cell starvation conditions prior to phage adsorption, host cells prepared as detailed above (Preparation of cell cultures), resuspended and incubated in S broth (1/2 of initial volume) at 37 °C during 17 h, were infected with phage B1 (m.o.i = 1). Adsorption experiments were carried out either in MRS or S broth as described previously.

### One-step growth curve

#### Influence of host cell starvation following phage infection

The one-step growth curves were performed in MRS broth as well as in each medium devoid of nutrients (S-C, S-N or S broth). Cell cultures subjected to the treatment described previously (Preparation of cell cultures) and resuspended in each medium (1/5 of initial volume) were infected with phage B1 at a m.o.i. of 1. After adsorption (30 min at 37 °C), phage-adsorbed bacteria were harvested by centrifugation (10,000 × g for 5 min), resuspended in a ten-fold volume of the corresponding medium added of CaCl_2_ (10 mM) and decimal dilutions of this suspension were carried out and incubated at 37 °C. At regular intervals, 100 μl aliquots of each dilution were collected for bacteriophage counts [17]. Results were related to initial phage counts and plotted against time.

On the other hand, similar experiments to those mentioned above were assayed when the favorable culture conditions were restored. Cell cultures prepared as aforementioned, resuspended in S or S-C broth and added of phage B1 (m.o.i. of 1) were subjected to adsorption test (30 min at 37 °C). Then, phage-adsorbed bacteria harvested by centrifugation (10,000 × g for 5 min) were diluted (decimal) in the corresponding medium (S or S-C) and incubated at 37 °C. After 30 and 60 min, carbon/nitrogen compounds or nitrogen compounds were added to the S or S-C broth, respectively. At regular intervals, 100 μl aliquots of each dilution were collected for bacteriophage counts. Similar experiments in starvation conditions (in S broth) during 5 and 17 h were performed. In this regard, cell cultures prepared as described previously, were resuspended in S broth and added of phage B1 (m.o.i. of 1). After adsorption (30 min at 37 °C), phage-adsorbed bacteria were collected, decimal dilutions were done in S broth and incubated at 37 °C. At 5 and 17 h of incubation, carbon/nitrogen compounds were restored, reaching the favorable culture conditions.

#### Influence of host cell starvation prior to phage infection

Cell cultures with the treatment described in “Preparation of cell cultures” were incubated at 37 °C for 1, 5 or 17 h in starvation conditions (S, S-C or S-N broth). After each starvation period, phage adsorption tests (30 min, 37 °C, m.o.i = 1) and the subsequent decimal dilutions of phage-adsorbed bacteria were performed in the corresponding medium (S, S-C or S-N) as well as in MRS broth, incubating at 37 °C. At regular intervals, 100 μl aliquots of each dilution were collected for bacteriophage counts [17]. Results were related to initial phage counts and plotted against time.

## Abbreviations

MRS: Man Rogosa Sharpe
S medium: Medium for starvation conditions in which both carbon and nitrogen sources were removed
S-C medium: Medium for starvation conditions in which nitrogen source was removed
S-N medium: Medium for starvation conditions in which carbon source was removed
